# Pre-existing Corticosteroid Use Predicts Worse Outcomes in Major Trauma

**DOI:** 10.7759/cureus.93002

**Published:** 2025-09-23

**Authors:** Gregory R Alfieri, Alison Thornton, Ilko Luque, Mark Mckenney

**Affiliations:** 1 Department of Medicine, Nova Southeastern University Dr. Kiran C. Patel College of Osteopathic Medicine, Fort Lauderdale, USA; 2 Trauma and Acute Care Surgery, HCA Florida Kendall Hospital, Miami, USA; 3 Surgery, University of South Florida, Morsani School of Medicine, Tampa, USA

**Keywords:** adverse outcomes, corticosteroids, glucocorticoids, in-hospital mortality, major trauma, national trauma data bank

## Abstract

Background and objective

Corticosteroids are one of the most commonly prescribed medications in the United States (U.S.), typically used for their benefits in autoimmune and inflammatory conditions. Major trauma (Injury Severity Score [ISS] >15) is a leading cause of death among young adults and is associated with increased mortality in the elderly population. In this study, we aimed to examine the association between pre-injury corticosteroid use and clinical outcomes in adults experiencing a major trauma event.

Methods

Patients (aged ≥18 years) admitted to U.S. Level 1 or 2 trauma centers for major trauma were selected retrospectively from the National Trauma Data Bank (2019-2021). Regression analysis was employed to determine the association between pre-existing corticosteroid use status and complications, including in-hospital mortality, adjusting for age, gender, ISS, and Revised Trauma Score (RTS).

Results

We identified 348,202 patients, of whom 0.8% (n = 2,733) had prior steroid use; 243,722 were males (70%), and the mean age was 51 years. Steroid use increased the odds of acute kidney injury (AKI) (adjusted odds ratio [aOR] = 1.4, p = 0.011), acute respiratory distress syndrome (ARDS) (aOR = 1.9, p<0.001), cerebrovascular accident (CVA) (aOR = 1.6, p = 0.003), unplanned intubation (aOR = 1.6, p<0.001), and in-hospital mortality (aOR = 1.5, p<0.001). Patients with pre-existing steroid use had a higher incidence of AKI (58 [2.1%] vs. 5,046 [1.5%]), ARDS (31 [1.1%] vs. 2,915 [0.9%]), CVA (41 [1.5%] vs. 2,788 [0.8%]), unplanned intubation (170 [6.3%] vs. 10,886 [3.3%]), and in-hospital mortality (428 [15.7%] vs. 37,014 [11.1%]) compared to their counterparts.

Conclusions

Corticosteroid usage before major trauma is a significant predictor of poorer outcomes and in-hospital mortality. Providers must factor this information into the management of patients on corticosteroid therapy who are at high risk of or have experienced major trauma.

## Introduction

Severe trauma is an increasing global problem that often affects younger, healthy adults [1]. Among those under 35 years of age, it is the leading cause of death and disability [2]. In the geriatric population, severe trauma results in a doubled mortality rate [2,3]. Major trauma has been defined as a traumatic event resulting in fatal or significant injury with deranged physiology, regardless of mechanism of injury, and/or is predicted to require significant treatment, such as ICU admission, surgical intervention, or the administration of blood products [4]. Trauma severity is commonly defined using the Injury Severity Score (ISS), an anatomic scoring system that assigns a numerical value (1-75) based on the number and severity of anatomic injuries [5,6]. The higher the ISS, the greater the degree of anatomical injury, with a score >15 defining major trauma [4,6]. An ISS >15 has historically been predictive of a 10% mortality rate, increased hospital length of stay, and cost [5].

Corticosteroids are one of the most widely prescribed drugs in the United States (U.S.) and have been used in almost all areas of medicine since their discovery in the 1930s [7-9]. Corticosteroids are synthetic analogs of natural steroid hormones produced by the adrenal cortex and include glucocorticoids and mineralocorticoids [9]. In clinical practice, the term “corticosteroids” usually refers to the glucocorticoids (GC) [8,9]. GCs are known to have immunosuppressive, anti-inflammatory, and vasoconstrictive effects [9]. They are also known to play a vital role in the treatment of inflammatory and autoimmune diseases, such as asthma, allergies, septic shock, rheumatoid arthritis, inflammatory bowel disease, and multiple sclerosis [8].

Despite their many benefits, widespread adverse effects of GCs have also been reported. Adrenal insufficiency (AI) can be induced through suppression of the hypothalamic-pituitary axis, hyperglycemia can occur due to increased insulin resistance, and osteopenia and osteoporosis can occur due to osteoclast stimulation. Psychiatric side effects include insomnia, anxiety, and even psychosis [9,10]. GCs also cause an increase in the risk of wound dehiscence and infection, an important consideration when dealing with patients who experience trauma [11,12]. In addition to these adverse effects, there is an argument that corticosteroids are overused and overprescribed. A 10-year-long study, conducted from 2009 to 2018, found that the average annual prevalence of oral corticosteroid (OCS) use was 6.8% in the U.S. and rose over the 10 years from 6.4 to 7.7% [7]. This rise was mostly due to increased short-term OCS use, most commonly for respiratory illnesses. Family practice and internal medicine physicians were the highest short-term OCS prescribers, and rheumatologists were the most common long-term prescribers. Elderly individuals and women were the most prescribed [7].

Using the National Trauma Data Bank (NTDB), we sought to compare differences in outcomes of major trauma in patients with pre-existing GC usage versus those without pre-existing usage. Our review of PubMed did not reveal any other study using NTDB to examine differences in severe trauma outcomes with associated GC usage. Currently published studies are single-institution studies analyzing chronic steroid use in trauma patients of all severity, citing a linkage between long-term use and impaired wound healing, compromised immune responses, and hindrance of bone healing, alongside the potential for AI during traumatic events [11]. Additionally, although not related to trauma, another large cohort study has demonstrated that baseline steroid use was associated with increased long-term risks of community-acquired infections and sepsis [13].

## Materials and methods

The NTDB was queried to extract data for adult patients (aged ≥18 years) admitted for trauma with an ISS >15 in U.S. level 1 or level 2 trauma centers, from 2019 through 2021. Patients’ age, gender, ISS, status on pre-existing steroid use, and in-hospital Revised Trauma Score (RTS) were collected. The RTS is a scale used worldwide to triage trauma patients and comprises the physiological patient parameters, systolic blood pressure (SBP), respiratory rate (RR), and the Glasgow Coma Scale (GCS) [14,15]. The outcomes were acute kidney injury (AKI), acute respiratory distress syndrome (ARDS), cerebrovascular accident (CVA), unplanned intubation, in-hospital mortality, cardiac arrest with cardiopulmonary resuscitation (CPR), deep surgical site infection (DSSI), deep vein thrombosis (DVT), extremity compartment syndrome, myocardial infarction (MI), organ/space SSI, pulmonary embolism (PE), and osteomyelitis.

Multiple logistic regression was used to explore how the pre-existing steroid use among patients was associated with each outcome previously mentioned, independently of age, gender, ISS, and RTS. Pre-existing steroid users are defined for the NTDB purposes as patients who require the regular administration of oral or parenteral corticosteroid medications within 30 days before injury for a chronic medical condition [16]. Statistical significance was defined as a p-value less than 0.05 with a 95% confidence interval (CI). The statistical software used for analysis was SPSS Statistics Version 29.0 (IBM Corp., Armonk, NY). This study received institutional review board exemption because of its retrospective design involving analysis of de-identified data.

## Results

Of the 2,843,852 adult patients (aged ≥18 years) in the NTDB, a total of 348,202 trauma patients with ISS >15 were included in this study; 243,722 (70.0%) were males, the mean age was 51 years, and 121,853 (35.0%) had suffered falls. The mean RTS was 6.9, and the mean ISS was 24.2 (Table 1). Out of 335,146 patients for whom pre-existing steroid use status was reported, 332,413 (99.2%) patients did not use steroids, while 2,733 (0.8%) patients met the criteria for steroid use. Pre-existing steroid use status was not reported for 13,056 (3.7%) out of 348,202 patients included in the study. In the steroid use group (n = 2,773 [0.8%]), the mean age was 67 years, and 1,532 (56.1%) were males (Table 1). In the non-steroid group (n = 332,413 [99.2%]), the mean age was 51 years, and 232,267 (69.9%) were males (Table 1). Table 2 summarizes the outcomes by pre-existing steroid use status.

**Table 1:** Characteristics of patients ^*^13,056 cases were not specified (3.7% of the total group) for steroid use status SD: standard deviation; RTS: in-hospital Revised Trauma Score; ISS: Injury Severity Score; AKI: acute kidney injury; ARDS: acute respiratory distress syndrome; CPR: cardiopulmonary resuscitation; SSI: surgical site infection; DVT: deep vein thrombosis; MI: myocardial infarction; PE: pulmonary embolism; CVA: cerebrovascular accident

Variables	Total, n = 348,202	Non-steroid group^*^, n = 332,413	Steroid group^*^, n = 2,733
Demographics			
Age, mean (SD)	51.3 (±20.8)	51.4 (±20.8)	67.3 (±15.7)
Gender, n (%)			
Female	103,059 (29.6)	98,748 (29.7)	1,190 (43.5)
Male	243,722 (70.0)	232,267 (69.9)	1,532 (56.1)
Not specified	1,421 (0.4)	1,398 (0.4)	11 (0.4)
Injury profile			
RTS, mean (SD)	6.9 (±1.8)	6.9 (±1.7)	7.3 (±1.3)
ISS, mean (SD)	24.2 (±9.4)	23.9 (±9.1)	22.3 (±7.0)
Mechanism of injury, n (%)			
Fall	121,853 (35.0)	117,655 (35.4)	1,796 (66.0)
Motor vehicle accident	112,164 (32.2)	107,349 (32.3)	591 (21.6)
Fire	1,629 (0.5)	1,543 (0.5)	10 (0.4)
Firearm	32,164 (9.2)	28,948 (8.7)	43 (1.6)
Cut/pierce	6,773 (1.9)	6,449 (1.9)	8 (0.3)
Hit by a car	22,770 (6.5)	21,286 (6.4)	57 (2.1)
Other	50,849 (14.6)	49,183 (14.8)	228 (8.3)
Body region injured, n (%)			
Head			
Yes	234,015 (67.2)	222,835 (67.0)	1,916 (70.1)
No	114,187 (32.8)	109,578 (33.0)	817 (29.9)
Face			
Yes	139,416 (40.0)	132,421 (39.8)	949 (34.7)
No	208,786 (60.0)	199,992 (60.2)	1,784 (65.3)
Neck			
Yes	22,993 (6.6)	21,591 (6.5)	108 (4.0)
No	325,209 (93.4)	310,822 (93.5)	2,625 (96.0)
Thorax			
Yes	190,901 (54.8)	181,481 (54.6)	1,191 (43.6)
No	157,301 (45.2)	150,932 (45.4)	1,542 (56.4)
Abdomen			
Yes	110,476 (31.7)	104,893 (31.6)	557 (20.4)
No	237,726 (68.3)	227,520 (68.4)	2,176 (79.6)
Spine			
Yes	122,656 (35.2)	117,536 (35.4)	911 (33.3)
No	225,546 (64.8)	214,877 (64.6)	1,822 (66.7)
Upper extremity			
Yes	148,604 (42.7)	141,512 (42.6)	1,093 (40.0)
No	199,598 (57.3)	190,901 (57.4)	1,640 (60.0)
Lower extremity			
Yes	155,436 (44.6)	148,110 (44.6)	1,104 (40.4)
No	192,766 (55.4)	184,303 (55.4)	1,629 (59.6)

**Table 2:** Outcomes evaluated by pre-existing steroid use status ^*^13,056 cases were not specified (3.7% of the total group) for steroid use status AKI: acute kidney injury; ARDS: acute respiratory distress syndrome; CPR: cardiopulmonary resuscitation; SSI: surgical site infection; DVT: deep vein thrombosis; MI: myocardial infarction; PE: pulmonary embolism; CVA: cerebrovascular accident

Outcomes, n (%)	Non-steroid group^*^, n = 332,413	Steroid group^*^, n = 2,733
AKI		
Yes	5,046 (1.5)	58 (2.1)
No	327,098 (98.4)	2,655 (97.2)
Not specified	269 (0.1)	20 (0.7)
ARDS		
Yes	2,915 (0.9)	31 (1.1)
No	329,245 (99.0)	2,682 (98.2)
Not specified	253 (0.1)	20 (0.7)
CVA		
Yes	2,788 (0.8)	41 (1.5)
No	329,365 (99.1)	2,673 (97.8)
Not specified	260 (0.1)	19 (0.7)
Unplanned intubation		
Yes	10,886 (3.3)	170 (6.2)
No	321,275 (96.6)	2,544 (93.1)
Not specified	252 (0.1)	19 (0.7)
In-hospital mortality		
Yes	37,014 (11.1)	428 (15.7)
No	295,399 (88.9)	2,305 (84.3)
Cardiac arrest with CPR		
Yes	9,348 (2.8)	67 (2.5)
No	322,825 (97.1)	2,646 (96.8)
Not specified	240 (0.1)	20 (0.7)
Deep SSI		
Yes	1,177 (0.4)	7 (0.3)
No	330,972 (99.5)	2,705 (98.9)
Not specified	264 (0.1)	21 (0.8)
DVT		
Yes	6,942 (2.1)	56 (2.0)
No	325,225 (97.8)	2,659 (97.3)
Not specified	246 (0.1)	18 (0.7)
Extremity compartment syndrome		
Yes	557 (0.2)	3 (0.1)
No	331,597 (99.7)	2,709 (99.1)
Not specified	259 (0.1)	21 (0.8)
MI		
Yes	933 (0.3)	13 (0.5)
No	331,218 (99.6)	2,699 (98.7)
Not specified	262 (0.1)	21 (0.8)
Organ/space SSI		
Yes	1,038 (0.3)	4 (0.1)
No	331,115 (99.6)	2,708 (99.1)
Not specified	260 (0.1)	21 (0.8)
PE		
Yes	3,186 (1.0)	27 (0.9)
No	328,968 (98.9)	2,686 (98.4)
Not specified	259 (0.1)	20 (0.7)
Osteomyelitis		
Yes	224 (0.1)	2 (0.1)
No	331,927 (99.8)	2,710 (99.1)
Not specified	262 (0.1)	21 (0.8)

Pre-existing steroid use was a significant predictor of AKI (adjusted odds ratio [aOR] [95% CI]): 1.4 (1.1 - 1.9), ARDS: 1.9 (1.3 - 2.7), CVA: 1.6 (1.2 - 2.3), unplanned intubation: 1.6 (1.4 - 1.9), and in-hospital mortality: 1.5 (1.3 - 1.6), independently of age, gender, ISS, and RTS (Tables 3, 4). Patients with pre-existing steroid use had a higher incidence of AKI (58 [2.1%] vs. 5,046 [1.5%]), ARDS (31 [1.1%] vs. 2,915 [0.9%]), CVA (41 [1.5%] vs. 2,788 [0.8%]), unplanned intubation (170 [6.3%] vs. 10,886 [3.3%]), and in-hospital mortality (428 [15.7%] vs. 37,014 [11.1%]) compared to their counterparts. We found no relationship between patients’ pre-existing steroid use status and cardiac arrest with CPR, deep SSI, DVT, extremity compartment syndrome, MI, organ/space SSI, PE, or osteomyelitis.

**Table 3:** Association between pre-existing steroid use and complications ^*^Statistically different from patients with steroid use after adjusting for age, RTS, and ISS (p<0.05) AKI: acute kidney injury; ARDS: acute respiratory distress syndrome; CVA: cerebrovascular accident; aOR: adjusted odds ratio; CI: confidence interval; RTS: in-hospital Revised Trauma Score; ISS: Injury Severity Score

Variables	AKI			ARDS			CVA			Unplanned intubation		
	aOR	95% CI	P-value	aOR	95% CI	p value	aOR	95% CI	p value	aOR	95% CI	p value
Steroid use	1.42	1.08, 1.87	0.011^*^	1.87	1.30, 2.67	<0.001^*^	1.64	1.19, 2.27	0.003^*^	1.60	1.36, 1.88	<0.001^*^
Age	1.01	1.007, 1.010	<0.001	0.99	0.990, 0.994	<0.001	1.01	1.01, 1.02	<0.001	1.02	1.017, 1.019	<0.001
RTS	0.93	0.92, 0.95	<0.001	0.90	0.88 0.92	<0.001	0.89	0.87, 0.91	<0.001	1.10	1.09, 1.12	<0.001
ISS	1.04	1.038, 1.043	<0.001	1.04	1.03, 1.04	<0.001	1.03	1.03, 1.04	<0.001	1.03	1.03, 1.04	<0.001

**Table 4:** Association between pre-existing steroid use on in-hospital mortality ^*^Statistically different from patients with steroid use after adjusting for age, RTS, and ISS (p<0.05) aOR: adjusted odds ratio; CI: confidence interval; RTS: in-hospital revised trauma score; ISS: injury severity score

Variables	In-hospital mortality		
	aOR	95% CI	P-value
Steroid use	1.46	1.30, 1.60	<0.001^*^
Age	1.03	1.032, 1.034	<0.001
RTS	0.61	0.61, 0.62	<0.001
ISS	1.05	1.046, 1.049	<0.001

## Discussion

Immunosuppression and infection

The immunosuppressive state induced by GCs is well documented [9,13,17]. This state is created by the prevention of leukocyte adhesion, activation of the complement cascade, and release of tumor necrosis factor, interleukin-1, and prostaglandins [18]. GCs also directly affect T-lymphocytes, suppressing delayed hypersensitivity reactions [17]. At supraphysiological doses, the immunosuppression from GCs can possibly lead to infections, such as pneumonia [19]. Prior studies have extensively linked steroids and infections [13,17,20], including findings of a doubled hazard ratio of serious infection and two to six-fold higher susceptibility to invasive viral and fungal infections [9,13,21]. Furthermore, other studies have focused on individuals with high-risk comorbidities, such as autoimmune diseases like rheumatoid arthritis, and targeted individual infections such as pneumonia, but not in the setting of major trauma [13]. Our study is the first to evaluate national outcomes in patients experiencing major trauma with pre-existing GC usage within 30 days before injury. Pre-existing GC use was a significant predictor of AKI, CVA, ARDS, unplanned intubation, and in-hospital mortality, after controlling for age, gender, ISS, and RTS (Tables 3, 4). No significant associations were found between pre-existing GC use and cardiac arrest with CPR, deep SSI, DVT, extremity compartment syndrome, MI, organ/space SSI, PE, or osteomyelitis.

Acute kidney injury

AKI is a common clinical syndrome that is characterized by abnormal renal function and structure and can increase the risk of in-hospital death, expense of care, and risk of early chronic kidney disease [22,23]. Our findings suggest a higher incidence of AKI in patients with prior GC usage compared to patients without prior GC usage among those with severe trauma (58 [2.1%] vs. 5,046 [1.5%]). A similar situation has been elucidated, where a patient developed AKI due to increased susceptibility to fungal infection, secondary to GC-induced immunosuppression [24]. Although this case did not occur in the setting of trauma, it may serve as an explanation for the increased incidence of AKI in severe trauma patients that was observed in our study. On the contrary, GCs may be a useful therapeutic strategy for septic AKI by reducing mitochondrial damage and apoptosis [25].

Furthermore, a single-center study (n=210) found that GC treatment in severe cases of coronavirus disease 2019 (COVID-19) is associated with a lower incidence of AKI [26]. Although no direct research was found regarding GC usage and severe trauma and AKI, this contradictory evidence suggests that the mechanisms of infection and trauma injury are affected differently by GC.

Cerebrovascular accidents

In non-trauma patients, preadmission use of GCs is associated with an increased 30-day mortality amongst patients with ischemic stroke, intracranial hemorrhage, and subarachnoid hemorrhage [19], as well as an increased 1-year cumulative risk of recurrent ischemic stroke [27]. The risk of venous thromboembolism is increased among GC users [28]. Our findings of increased CVA incidence in patients with prior GC compared to patients without prior GC usage among those with severe trauma (41 [1.5%] vs. 2,788 [0.8%]) align with previously documented findings [19,27]. One possible explanation includes GC-induced adrenal suppression that compromises the cortisol response to critical illness, such as stroke [19]. It has also been proposed that long-term GC use is associated with increased risk of atrial fibrillation, diabetes mellitus, and hypertension, which are all strong predictors of mortality in stroke [19]. We theorize that patients with prior GC usage are more likely to be plagued with previously stated medical conditions, thus are predisposed to increased CVAs when severe trauma is experienced.

Unplanned intubation and ARDS

The results of our study revealed that patients with pre-existing GC usage had a significantly increased risk of unplanned intubation. A previous study, although unrelated to trauma, found that patients who underwent unplanned re-intubation within 24 hours after surgery also had an increased risk with GC usage [29].

ARDS is a serious condition that can cause severe inflammation and damage to the lungs, leading to difficulty breathing and hypoxemia. Regarding GCs, while therapeutic steroid usage decreases mortality risk in patients with ARDS, when used prophylactically, it can increase the risk of developing ARDS and the risk of death [30]. Our study found that the incidence of ARDS was higher in patients with pre-existing GC usage compared to patients without pre-existing GC usage following a severe trauma event (31 [1.1%] vs. 2,915 [0.9%]). Therefore, while therapeutic GCs can be helpful to reduce mortality risk in patients with ARDS who have not experienced a trauma event [30,31], it is also vital to understand that GCs can increase the chance of developing ARDS following trauma.

Cardiovascular disease risk

Glucocorticoid risk is associated with a higher cardiovascular disease (CVD) risk, including hypertension, hyperglycemia, and obesity [17,32]. Furthermore, the rate of CVD events appears to be significantly higher in patients prescribed high GC doses (≥7.5 mg/day of prednisone or equivalent) versus those who had not received GCs [17,32]. A small increased risk of acute myocardial infarction (MI) has also been reported with the use of oral corticosteroids, with a greater risk amongst users of high corticosteroid doses [33]. Our results contrast with these results when in the setting of a major trauma event, as we found no significant increase in the incidence of acute MI with pre-existing GC use.

Future implications

Although the deleterious side effects of GCs have been widely reported, there is still limited literature on the specific relationship between outcomes from severe trauma and pre-existing GC use. One single institution study (n = 18), looking at all trauma severity levels and steroid usage, found patients had impaired wound healing, compromised immune responses, and hindrance of bone healing, and potential adrenal insufficiency during traumatic events [11]. Further complications included orthopedic fractures and ICU admissions. Our study differed in that we had a large sample size from NTDB (n = 2,733 steroid users), and we only included severe trauma patients (ISS >15). This study found increased mortality as opposed to low mortality previously seen in literature (5.6% of total patients expired) [11].

With the large data set and the severity of the outcomes measured, particularly mortality, we hope to bring awareness to this matter and allow clinicians to make the proper changes in management in anticipation of worse outcomes in patients on GCs who experience severe trauma. With the widespread usage of GCs in the United States [7-9], it is important to understand the impact these findings can have on the clinical management of severe trauma patients, as certain outcomes can be anticipated and prepared for before they occur. Severe trauma alone is associated with poor outcomes, and the presence of pre-existing GC usage will both complicate the management of these patients and perpetuate poorer outcomes.

Limitations

There are several potential limitations of the study. One limitation is that it is unknown the true length of time and dosage of GC patients were placed on before experiencing trauma. The variation in dosage and duration could have affected results due to the varying degree of adverse effects GC endured by the body at the time of trauma. Another limitation is that high-risk individuals, such as the elderly, are commonly placed on steroids. This group could have disproportionately impacted the outcome of the results, such as mortality. Further, the past medical history of the patients was not factored into the statistical analysis and could have affected outcomes. Lastly, the results of this study should be understood with the limitations of database research in mind. This includes improper coding, resulting in over- or under-documentation of diseases, missing data, or inaccurate data that can bias results [34,35].

## Conclusions

Pre-existing steroid use in patients experiencing major trauma (ISS >15) is associated with significantly worse outcomes, including associated increases in AKI, ARDS, CVA, unplanned intubation, and increased mortality. These findings highlight the need for a more detailed consideration of steroid therapy in patient populations who are susceptible to severe trauma. Further research is needed to determine the dosage and duration of GC, which carries the highest risk of poor outcomes, as well as to explore potential therapeutic strategies to mitigate the risks associated with steroid use in this population. Clinicians must be vigilant when handling patients with major trauma, particularly those with prolonged steroid therapy, and consider strategies to minimize the risks of adverse outcomes.
